# Ethnic and Socio-Economic Variations in Comorbidity and Mortality in Cancer Survivors: A UK Population-Based Observational Study

**DOI:** 10.3390/cancers17060983

**Published:** 2025-03-14

**Authors:** Tahania Ahmad, Abu Z. M. Dayem Ullah, Claude Chelala, Stephanie J. C. Taylor

**Affiliations:** 1 Wolfson Institute of Population Health, Queen Mary University of London, London E1 4NS, UK; s.j.c.taylor@qmul.ac.uk; 2 Barts Cancer Institute, Queen Mary University of London, London EC1M 6BQ, UK; d.ullah@qmul.ac.uk (A.Z.M.D.U.); c.chelala@qmul.ac.uk (C.C.)

**Keywords:** multimorbidity, comorbidity, cancer survivors, morbidity, ethnicity, socio-economic status, mortality, IMD, SES, United Kingdom, UK

## Abstract

The population of cancer survivors is increasing rapidly in the UK. Little is known about the variation in comorbidity and mortality by ethnicity and socio-economic condition in this population. This study explores these variations using primary care data from the Clinical Practice Research Datalink (CPRD) and linked secondary care data. The prevalence of multimorbidity and risk of mortality were calculated for Asian, Black, and Other ethnic and socio-economic groups in England, consisting of 333,226 cancer survivors across 28 cancer types. Odds ratios and hazard ratios were calculated using the White and most affluent groups as references and adjusted for age, sex, BMI, and smoking status. Stratified mortality analysis was conducted for survivors of the six common cancers in the UK: breast, prostate, colorectal, bladder, cervical, and lung. Compared to White cancer survivors, survivors of all other ethnic groups had a statistically significant higher prevalence of type 2 diabetes (Asian adjusted odds ratio (OR) 4.61 (4.02–5.28), Black OR 1.87 (1.52–2.30), and Other OR 2.06 (1.64–2.59)). However, they had lower prevalences of depression and anxiety. Asian survivors exhibited the highest overall prevalence of comorbidity. Black survivors had the worst survival (adjusted hazard ratio (HR) 1.48 (1.38–1.59)) for all cancers combined, as well as for breast, prostate, colorectal, and cervical cancers. Black breast cancer survivors face a particularly high mortality risk (HR 1.78 (1.52–2.10)) compared to Whites. Asian survivors had higher mortality for all cancers combined (HR 1.31 (1.23–1.39)) and specifically for lung cancer (HR 1.81 (1.44–2.28)). The Other ethnic group had a significantly increased risk of mortality in cervical cancer (HR 1.90 (1.19–3.03)). The risk of mortality increased with worse socio-economic conditions, regardless of ethnic group. Cancer survivors of non-White ethnicity and poorer socio-economic background in the UK have worse outcomes in terms of increased prevalence of multimorbidity and mortality compared to White survivors. These findings indicate the need to comprehend the underlying reasons for these disparities and to assess the implications for cancer services, patient experience, and overall outcomes.

## Simple Summary

This study examines the health challenges and survival rates of cancer survivors from different ethnic and socio-economic backgrounds in the UK. It analysed data from over 333,000 cancer survivors across 28 types of cancer. The findings showed that compared to White cancer survivors, people from Asian, Black, and Other ethnic groups had a higher prevalence of type 2 diabetes but a lower prevalence of depression and anxiety. Asian survivors had the highest overall health problems, while Black survivors had the highest death rate, particularly for breast, prostate, and colorectal cancers. Asian survivors had the highest risk of dying from lung cancer. Additionally, death rates worsened with increasing social deprivation, regardless of ethnicity. The findings highlight the need for tailored healthcare strategies for cancer survivors from diverse ethnic backgrounds.

## 1. Introduction

The global burden of cancer is rapidly increasing, with 19.3 million new cases in 2020, expected to rise to 28.4 million in 2040 [1]. In the United Kingdom (UK), someone is diagnosed with cancer every two minutes, and one in four deaths is cancer related [2,3,4]. Cancer risk and outcomes vary by ethnicity [5]. With increasing ethnic diversity in the UK, understanding ethnicity-related factors influencing cancer outcomes is crucial [3,4]. National data on cancer survival by ethnic group are limited [3,4]. Existing evidence suggests that Black African and Caribbean populations are more likely to be diagnosed with advanced-stage breast or prostate cancers and have lower survival rates compared to their White British counterparts [6,7,8,9].

There has been much discussion in the literature on the complex and interrelated factors, such as biological and environmental factors, that contribute to ethnic disparities in cancer-related outcomes [4,10,11,12]. These disparities are also influenced by socioeconomic inequalities, which restrict access to high-quality care, screening/prevention, prompt diagnosis, and treatment [5,11]. Socio-economic status (SES) influences cancer incidence, survival, and comorbidities in various ways. Cancer risk and lower SES share some common risk factors such as smoking, poor nutrition, occupational exposures, obesity, and physical inactivity. Greater educational attainment and SES are both associated with health-conscious behaviours such as better diets, increased physical activity, and participation in health promotion and screening programs [13]. Access to and use of healthcare services is also influenced by SES. Available evidence from developed countries shows consistent relationships between cancer and SES [14,15,16]. Cancers of the stomach, liver, lung, upper gastro-intestinal tract, and cervix have historically been more common in socially disadvantaged groups, whereas breast and ovarian cancer are more common in higher SES groups [13,17].

The presence of comorbidity (defined here as having two or more long-term conditions, including cancer) potentially affects the diagnosis of cancer, the treatment received, and a person’s healthcare needs. Some patients may receive an earlier diagnosis because of frequent interactions with healthcare providers, while others may receive a delayed diagnosis due to symptoms shared with a comorbid condition, as in the case of lung cancer and chronic obstructive pulmonary disease (COPD) [18]. Numerous long-term side effects, including chronic pain and fatigue, sexual dysfunction, anxiety, depression, and lymphedema, have been linked to cancer treatment [19,20]. It is also less likely that cancer patients with multiple comorbidities will receive curative care [18].

There is very little published research in the UK exploring comorbidity in cancer survivors by ethnicity and SES [21]. Previous studies on this topic have focused on a single cancer, a single ethnicity, or a small group of comorbidities [22,23]. This study investigates ethnic and socio-economic variation in comorbidity and mortality in cancer survivors, adjusting for demographic factors such as age, sex, body mass index (BMI), and smoking. The scope of our study encompasses 28 cancers in the UK (with a special focus on 6 common cancers), 11 different ethnic groups (collapsed to 4 broad ethnic groups for comorbidity analysis), and 15 comorbidities.

## 2. Materials and Methods

### 2.1. Study Design and Data Sources

This study presents a secondary analysis of a broader research project titled “Characterisation of multimorbidity clusters and trajectories using data-driven approaches in a nationally representative population” [24]. The data utilised in this study were sourced from the Clinical Practice Research Datalink (CPRD) GOLD [25]. The primary care data from the CPRD were linked with death registration records from the Office of National Statistics (ONS) and individual postcode-based indices of multiple deprivation data (IMD).

### 2.2. Participants

Our study population comprised all patients permanently registered in any English General Practice that met ‘up to standard’ CPRD GOLD data quality criteria between 1 January 2010 and 31 December 2020 and had at least one long-term condition (from a set of 204 conditions) recorded during this time frame. In total, 28 cancers were included in the list of 204 conditions, forming the cohort of this analysis. A comprehensive account of the selection procedure for the 204 conditions is accessible through an open GitHub repository [26].

We selected cancer survivors who had a diagnosis of any of the 28 cancers (Table A1 in Appendix A), were over the age of 18 at diagnosis, and had survived cancer for at least two years. There is no universal definition of cancer survivorship; however, a two-year cut-off is one of the most commonly used [21]. The two-year cutoff was chosen to include participants who had completed primary treatment for cancer and to exclude those with terminal cancer, as the focus of this paper is “cancer survivors”. Cancer diagnosis was defined as the first instance of the code of selected cancer in CPRD.

### 2.3. Study Variables

Cancer types: Data from all 28 cancer types are included for “all-cancer combined” analyses. Stratified mortality analysis was conducted for the six common cancers in the UK: breast, prostate, colorectal, bladder, cervical, and lung cancer.

Comorbidity: The 15 most prevalent comorbidities in cancer survivors (all cancers combined), as recorded in the CPRD data, were chosen based on our previous work [27]. These comorbidities in order of prevalence were hypertension, painful conditions, osteoarthritis, depression, constipation, dermatitis, hearing loss, coronary heart disease (CHD), anxiety and phobia, chronic kidney disease (CKD), anaemia, atrial fibrillation, asthma, obesity, and type 2 diabetes. Any record of these 15 comorbidities in the CPRD data was included in the analysis, regardless of whether it arose before or after cancer. Data on comorbidities were extracted from the CPRD using medical codes relating to 204 long-term conditions, as described by Eto and colleagues [24].

Ethnicity: The patients’ ethnicity was derived from the CPRD record. We extracted all ethnicity records from the CPRD using medical codes, read codes, and ethnicity-related terms in the CPRD look-up files. Eleven ethnic groups were included: White, Black African, Black Caribbean, Black Other, Chinese, Other Asian, Bangladeshi, Indian, Pakistani, Mixed, and Other ethnic groups. We collated the ethnicities into five major ethnic categories according to ONS grouping used in census data: White, Black or Black British (Black African, Black Caribbean, and Black Other, referred to as Black in the rest of the paper for simplicity), Asian (Indian, Bangladeshi, Pakistani, Chinese, and other Asians), Mixed, and Other [28].

Other variables: The patients’ age, sex, smoking, and mean BMI were identified from the CPRD data. Age was calculated by subtracting year of birth from year of death, or the censoring date (31 December 2020). Smoking was categorised into never smoker, current smoker, or former smoker, based on the last known status. Mean BMI (mean of all available BMI recordings in each individual’s medical record) was preferred over last recorded BMI because unexpected weight loss is a common cancer symptom [29]. Deprivation was measured using quintiles of the 2015 IMD, available via CPRD linkage, and corresponding to an individual’s address during the latest GP registration. For mortality, “all-cause mortality” from linked ONS data was used.

### 2.4. Statistical Analysis

The purpose of this study was to identify any variation in comorbidity and mortality by ethnicity and socio-economic status (indicated by IMD) in cancer survivors. To explore variations in comorbidity prevalence by ethnicity, we fitted multivariable logistic regression models, adjusting for age, sex, mean BMI, and smoking status to find the odds of having a particular comorbidity. We applied clustered standard errors to account for potential clustering by primary care practice [30]. The primary outcome of this model was the presence or absence of the specified comorbidity (from the fifteen selected comorbidities). Individual models were created for the six cancer types to understand how comorbidity varied in each cancer by ethnicity and IMD. Crude and adjusted odds ratios for each ethnic group were calculated. The Mixed ethnic group was excluded as the numbers were too low when stratified by cancer and comorbidities. Participants with less than two years of follow-up data, no information on smoking, BMI, or IMD were excluded from the analyses. However, a sensitivity analysis was conducted using multiple imputations for ethnicity for comparison purposes.

To calculate the risk of mortality by ethnicity and IMD, we fitted separate Cox proportional hazard regression models for each of the six cancers, adjusting for age, sex, latest smoking status, comorbidity, mean BMI, and ethnicity/IMD (whichever is appropriate). The time since cancer diagnosis to death or end of study was taken as the timescale. Crude and adjusted hazard ratios were calculated.

For all multivariable regression models, the reference groups used were White ethnicity, most affluent socio-economic quintile group (IMD1), and never smoker. BMI and age were entered as continuous variables. Data were presented as mean (SD) for continuous data, proportion (%) for categorical data, or as adjusted odds ratios (OR) or adjusted hazard ratios (HR) with 95% confidence intervals. All analyses were performed in R version 4.2.0.

## 3. Results

**Figure 1:**
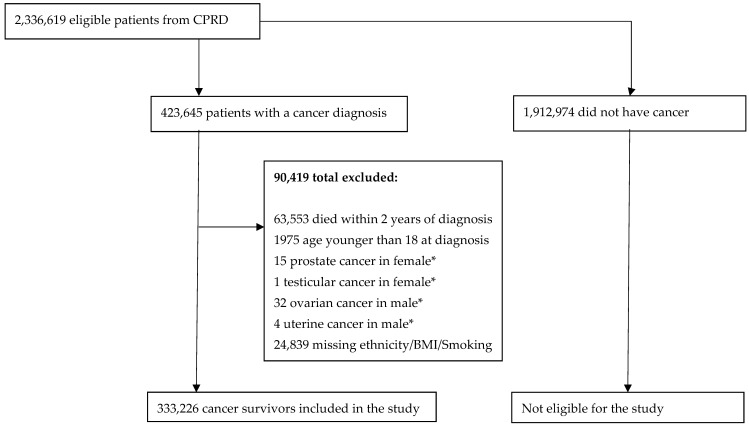
Study cohort profile. BMI = Body mass index. IMD = Index of multiple deprivation. * Not possible to determine if reporting error or trans-sex individual is omitted.

The overall cohort consisted of 423,645 cancer patients from 28 cancer types, of whom 333,226 met the inclusion criteria (Figure 1). Table 1 shows the characteristics of these patients. In total, 96.2% cancer survivors were of White ethnic background, followed by Asians (1.6%) and Blacks (1.1%). There were more female cancer survivors than male across all ethnicities, and most cancer survivors were above the age of 60. More than a quarter of those in the White survivors belonged to the most affluent category (IMD1) (26.3%), similar to Other survivors (23.2%). In comparison, only 6.8% of the Black survivors were in the most affluent category, while 36.3% were in the most deprived category (IMD5). Among Asians, the proportion of survivors in each IMD group had less variability (17.7–21.8%). Whites had the highest percentage of former smokers (33.9%). The average age at death was seven years younger for the Black survivors compared to the Whites. Among the cancer survivors, the most common cancer across all ethnic groups was breast cancer (>20%), except among the Black survivors, where it was prostate cancer (22.9%). The proportion of Asian survivors with breast cancer (25.2%) was higher than that of the other ethnic groups.

**Figure 2:**
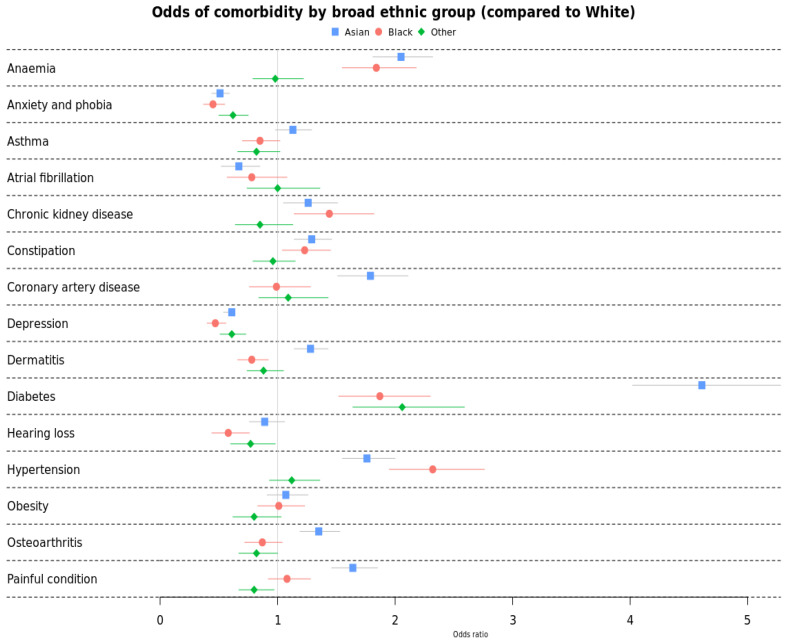
Adjusted odds ratio of prevalence of comorbidity by ethnicity in cancer survivors. Adjusted for age, sex, IMD, BMI, and smoking status.

Figure 2 below presents the adjusted odds ratio for individual comorbidity for all cancer survivors (from all-cancers combined) by broad ethnic groups, with White ethnic group as the reference (Table A2). Asian survivors had significantly higher odds of 9/15 comorbidities compared to Whites: type 2 diabetes (OR 4.61 (4.02–5.28), anaemia (OR 2.05 (1.81–2.32)), coronary artery disease (OR 1.79 (1.51–2.11)), hypertension (OR 1.76 (1.55–2.00)), painful conditions (OR 1.64 (1.46–1.85)), osteoarthritis (OR 1.32 (1.19–1.53)), constipation (OR 1.29 (1.14–1.46)), dermatitis (OR 1.28 (1.14–1.43)) and chronic kidney disease (OR 1.26 (1.05–1.51)). However, Asians had lower odds of depression, anxiety and phobia, and atrial fibrillation.

Black survivors had higher odds of only 5/15 comorbidities compared to Whites: hypertension (OR 2.32 (1.95–2.76)), diabetes (OR 1.87 (1.52–2.30)), anaemia (OR 1.84 (1.55–2.18)), CKD (OR 1.44 (1.14–1.82)) and constipation (OR 1.23 (1.04–1.45)). They had significantly lower odds of dermatitis, hearing loss, depression, and anxiety/phobia. The Other ethnic group had significantly higher odds of only 1/15 comorbidity—diabetes (OR 2.06 (1.64–2.59)).

**Figure 3:**
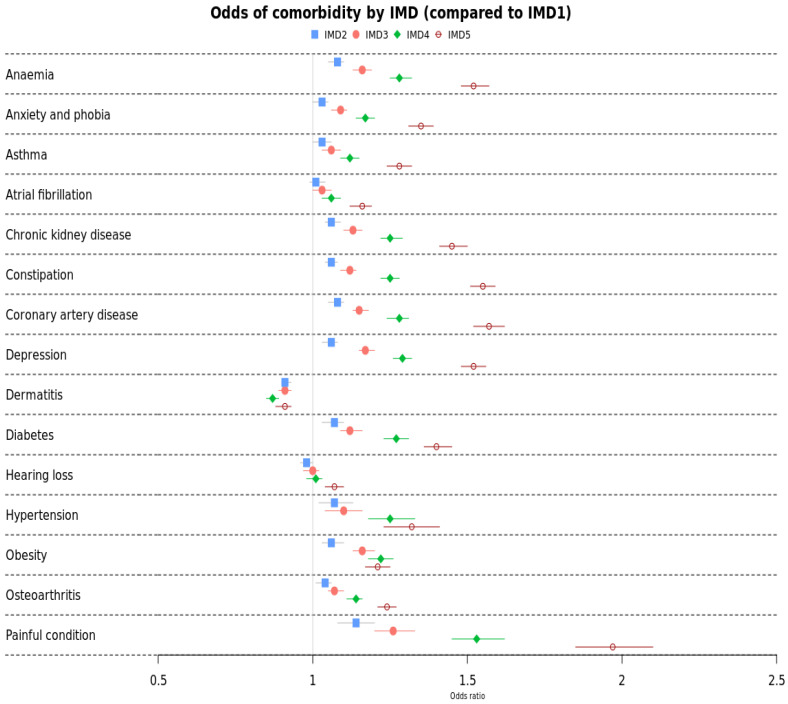
Adjusted odds ratio of prevalence of comorbidity by IMD in cancer survivors. Adjusted for age, sex, ethnicity, BMI, and smoking status.

Figure 3 below presents the adjusted odds ratio of comorbidities by IMD, with IMD1 (most affluent) being the reference point (Table A3). There was a clear socio-economic gradient with increasing odds of comorbidity as IMD worsened for all comorbidities except dermatitis and hearing loss. There was a particularly strong socio-economic gradient (OR > 1.40 for IMD5) in painful conditions, depression, constipation, coronary artery disease, and anaemia.

### 3.1. Morality by Ethnicity

**Figure 4:**
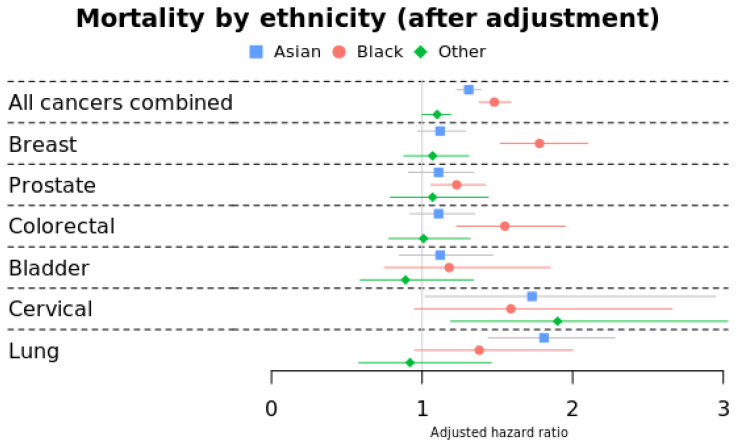
Risk of morality by ethnicity stratified by cancer site (adjusted for age, sex, smoking status, IMD, and BMI). Reference group: Whites.

Black and Asian cancer survivors exhibited worse survival than Whites across all cancer types, even after adjusting for age, sex, IMD, BMI, and smoking status (Figure 4 and Table A4). Black survivors had the worst survival (HR 1.48 (1.38–1.59)) for all cancers combined, as well as breast, prostate, colorectal, and cervical cancers. Black breast cancer survivors faced a particularly high mortality risk (HR 1.78 (1.52–2.10)) compared to White survivors. Asian survivors showed slightly lower mortality than Black survivors for all cancers combined (HR 1.31 (1.23–1.39)) but had higher risk of death for lung cancer (HR 1.81 (1.44–2.28)) and cervical cancers (HR 1.73 (1.02–2.95)). The Other ethnic group had a significantly increased risk of mortality in cervical cancer (HR 1.90 (1.19–3.03)).

### 3.2. Mortality by IMD

**Figure 5:**
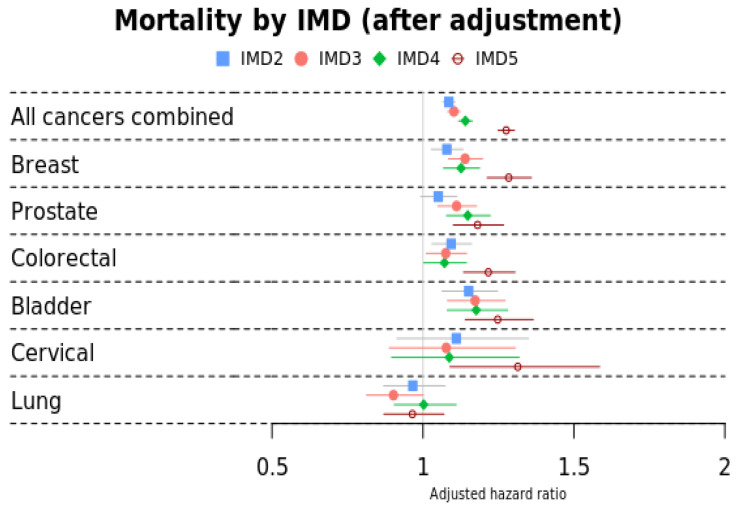
Mortality by IMD stratified by cancer site adjusted for age, sex, ethnicity, smoking status, and BMI. Reference group: IMD1 (most affluent).

When comparing mortality by SES (indicated by IMD), we found a clear socio-economic gradient in mortality for all cancers combined with mortality risk increasing as IMD worsened (Figure 5 below and Table A5). This pattern persisted after adjusting for ethnicity, age, sex, smoking status, and BMI. There was a strong gradient across the SES groups for prostate and bladder cancer survivors. For colorectal and cervical cancer survivors, we noted a much worse survival only for IMD5 (least affluent). For lung cancer survivors, the results were not statistically significant. 

## 4. Discussion

Research exploring associations between comorbidity and ethnicity among cancer survivors in the UK is very limited. This study identified disparities in comorbidity and mortality in cancer survivors (defined as people who had survived their first diagnosis of cancer by at least 2 years) by ethnicity and socio-economic status. We observed notable differences in the prevalence of comorbidity among ethnic groups. Asian cancer survivors exhibited the highest overall comorbidity prevalence compared to Whites, showing increased odds for 9 out of 15 comorbidities including type 2 diabetes, anaemia, coronary artery disease, hypertension, painful conditions, osteoarthritis, constipation, dermatitis, and chronic kidney disease. The prevalence of anaemia was particularly high in Asian survivors. This association may be influenced by the fact that Asians are known to have a higher prevalence of anaemia compared to non-Asians [31] and 40–60% of cancer patients are afflicted by anaemia [32].

Black cancer survivors had higher odds of five comorbidities compared to Whites—hypertension, type 2 diabetes, anaemia, chronic kidney disease and constipation. Many studies have reported a higher prevalence of hypertension among Black and South Asians compared to Whites [33], and this holds true for cancer survivors also. The Other ethnic survivors consistently exhibited higher odds of type 2 diabetes. All broad ethnic groups had significantly higher odds of type 2 diabetes compared to Whites. The association between type 2 diabetes and cancer is well established [34,35,36,37]. A consensus report published by the American and European Diabetes and Oncology Association agreed that most evidence suggests a strong link between diabetes and breast, colorectal, endometrial, liver, and pancreatic cancers, and that the likely pathogenesis of the link is due to hyperinsulinemia, hyperglycaemia, inflammation, and possibly diabetes [36]. This association is even more pronounced for minority ethnic groups and the economically disadvantaged [38,39]. Existing research reports a higher comorbidity prevalence in non-White cancer survivors [21,40,41,42,43,44,45,46] and in the general population compared to Whites [47]. We found that other ethnic groups had a lower prevalence of depression and anxiety compared to Whites, whereas an Australian study found higher risks of these comorbidities in migrant populations [44]. Comorbidities, except hearing loss, dermatitis, and atrial fibrillation, showed a clear socio-economic gradient, and comorbidity prevalence increased with deprivation.

All non-White survivors had higher mortality risks compared to White survivors. Significantly higher mortality was seen in Asian lung and cervical cancer survivors, in breast, prostate, and colorectal cancer survivors of Black ethnicity, and in cervical cancer survivors of the Other ethnic group. There is a substantial association between socio-economic status and mortality across all cancers, persisting after adjusting for confounding factors. The least affluent group consistently exhibited the highest mortality rates. For lung cancer survivors, no discernible significant pattern emerged. This observation may be attributed to the cohort’s composition (selection bias), consisting of individuals who survived cancer for two or more years. Notably, lung cancer tends to be diagnosed at later stages among those who are socio-economically deprived, and this subset is more likely to die within two years of diagnosis, according to previous research [48].

However, all-cause mortality was significantly worse in all non-White ethnic groups. Compared to White survivors, we found that Blacks survivors were 48% more likely, Asians survivors were 31% more likely to have died, and the Other ethnic group survivors were 10% more likely. The National Cancer Intelligent Network report confirms our finding of a higher prevalence of prostate cancer and significantly higher mortality among Black breast cancer patients (7). Asians were more likely to die from lung cancer than other ethnicities; the most plausible reason cited previously was late stage at presentation [49].

To our knowledge, this is one of the few studies in the UK to explore a large number of comorbidities in broad ethnic groups of cancer survivors. A major strength of this study is the size and breadth of the linked data sources and the use of a robustly defined method to select comorbidities and included cancers. A systematic and comprehensive procedure was utilised for comorbidity and cancer coding and made available for public use [50]. The benefits of employing CPRD GOLD data connected to secondary care data sources, notably for cancer, have been shown by numerous validation studies [51,52,53]. The significance of this is demonstrated by the variability observed in the association of cancer types and comorbidities, showing how important variations can be masked when comorbidities or cancers are grouped together. Using linked data allowed us to adjust for smoking, BMI, IMD, and ethnicity as risk factors for developing comorbidity, enabling us to determine the impact of these factors on prevalence estimates for individual comorbidities. For the purpose of increased power and simplicity, higher-level ethnic categorisation was used for comorbidity analysis [54]. We used “all-cause mortality” as the outcome rather than cancer-specific mortality because cancer-specific death estimates may be invalid if there is misclassification of the cause of death [55] and cancer survivors are more likely to die from other causes rather than the cancer itself [56]. Moreover, a recent paper found that all-cause mortality is a better choice for studies making group comparisons such as this one [57]. Our approach elucidates which comorbidities are associated with very high odds of poor outcomes in different ethnic groups. The identification of these offers a basis for creating better treatment pathways for the management and possible prevention of comorbidity in cancer survivors. 

There are some limitations to this study; we were unable to have linkage to cancer registry data to confirm the date of diagnosis of cancer, although a prior study [52] found minimal differences in the date of cancer diagnosis between CPRD and cancer registry data. We did not have information on cancer staging and cancer treatments, such as chemotherapy and radiotherapy, which precluded us from examining the correlation between these factors and comorbidity. We had access to hospital records but did not use it for this study, as comorbidities are more comprehensively recorded in primary care [58]. Residual confounding may exist due to the possibility of additional unmeasured factors in our analysis (e.g., educational level and aetiological factors). 4% of cancer survivors were excluded due to missing ethnicity recording; however, our sensitivity analysis with multiple imputation for ethnicity found minimal differences in effect sizes before and after imputation; thus, we presented a complete case analysis here. In total, 96% of our cancer survivors’ cohort was White; thus, the findings may not be generalisable to a more ethnically diverse population. The higher representation of the White population in the study is largely due to the UK population structure; however, cancer incidence is also higher among Whites [4]. Another limitation is that the Other group was a very mixed group in terms of ethnicities included, so caution should be exercised in drawing conclusions from this group.

The earlier mortality in all other ethnic groups compared to the Whites can be attributed to lifestyle factors, biological factors, socio-economic status, structural issues, access to screening/healthcare, differing cultural norms and languages, existing comorbidities, and many other factors [48]. In comparison to Whites, Asian and Black patients in this study were younger and resided in more impoverished areas, reflecting the demography of major ethnic groups in the UK [24]. Although socio-economic disparities may contribute to these inequalities in comorbidity and mortality, it does not fully explain differences in some cancers, e.g., higher prevalence of prostate cancer in Black men. Even after considering socio-economic status, environmental factors, and healthcare access, African American men still face a higher risk and mortality rate for prostate cancer, indicating the influence of other factors [59,60]. Another interesting observation in this study was that Black cancer survivors had a lower prevalence of comorbidities compared to Whites, yet higher mortality. This may indicate undiagnosed comorbidities.

The strong socio-economic gradient in comorbidity and mortality across all cancer types may suggest barriers to access to healthcare and lower awareness of cancer among the more deprived populations. Prior research indicates that compared to those living in more affluent areas, people with comorbidity who live in more impoverished areas may receive lower-quality healthcare, as evidenced by shorter consultation times, less patient-centeredness, and lower perceived GP empathy [61,62]. A recent study found that where inequalities exist, the adverse cancer outcomes among Asian and Black patients are unlikely to be solely due to poor diagnostic procedures [63]. In this study, we found that non-White cancer survivors had a significantly higher prevalence of type 2 diabetes, hypertension, and anaemia and a lower prevalence of depression, anxiety, and atrial fibrillation. The increased comorbidity linked to lower socio-economic status may also play a role in diminishing cancer survival. More funding and research are required to tackle these comorbidities in minority ethnic groups and deprived populations, and to tackle depression and anxiety in Whites. It necessitates intervention by clinical and public health systems to address the upstream determinants of health that contribute to these disparities and subpar outcomes in cancer survivors.

The prevalence of cancer survivors is likely to increase in the UK in the near future due to continued improvement in cancer treatments, increasing early diagnosis and the ageing demographic [64]. Consequently, there is a pressing need to develop and evaluate interventions focused on enhancing outcomes for cancer survivors. Ethnic minorities have worse mortality rates than Whites. A plausible reason is lower cancer awareness and poor uptake of cancer screening in these groups. This is particularly true for breast cancer among Asian women in the UK [65]. Disparities in the utilisation of cancer screening procedures exist, even in countries with universal insurance coverage [66,67]. A recent review underscores that socioeconomic inequalities in screening tend to be reduced in countries with comprehensive population-based screening programs compared to those relying solely on opportunistic screening [68]. Existing evidence supports specific strategies, such as offering free tests, eliminating geographical barriers, involving primary care physicians more actively, and employing individually tailored communication as effective means to enhance screening accessibility among lower socioeconomic groups [69]. This reinforces the notion that preventive measures and interventions may necessitate innovative and tailored approaches to effectively reach the most vulnerable subgroups and socioeconomically deprived segments of the population.

## 5. Conclusions

Current UK guidelines to address and manage cancer patients [70] do not yet include management of comorbidity, nor do they include guidance on equity in healthcare for cancer survivors and the distribution of resources based on need. Public health initiatives aimed at improving outcomes for cancer survivors should focus on screening uptake, improving cancer awareness, lowering comorbidity, and including more targeted guidelines for minority populations and those with lower SES. Further research is required to investigate the reasons for the disparities in outcome among cancer survivors, with potential explanations including past migration patterns, socioeconomic composition of the groups, health-related behaviours, and clinical and biological factors.

## Figures and Tables

**Table 1:** Characteristics of cancer survivors by broad ethnic groups.

Characteristics (n, %)	White (n = 320,413; 96.2%)	Asian (n = 5379; 1.61%)	Black or Black British (n = 3539; 1.06%)	Other (n = 2840; 0.32%)	Mixed (n = 1055; 0.85%)
**Sex**					
Female	180,832 (56.4%)	3247 (60.4%)	1940 (54.8%)	1728 (60.8%)	658 (62.4%)
Male	139,581 (43.6%)	2132 (39.6%)	1599 (45.2%)	1112 (39.2%)	397 (37.6%)
**Age groups, years**					
(18,30]	809 (0.3%)	33 (0.6%)	15 (0.4%)	17 (0.6%)	8 (0.8%)
(30,45]	13,155 (4.1%)	612 (11.4%)	335 (9.5%)	306 (10.8%)	177 (16.8%)
(45,60]	42,181 (13.2%)	1240 (23.1%)	1079 (30.5%)	686 (24.2%)	308 (29.2%)
(60,75]	101,532 (31.7%)	1947 (36.2%)	1025 (29.0%)	1019 (35.9%)	307 (29.1%)
(75,90]	131,497 (41.0%)	1415 (26.3%)	988 (27.9%)	684 (24.1%)	216 (20.5%)
90+	31,239 (9.7%)	132 (2.4%)	97 (2.7%)	128 (4.5%)	39 (3.7%)
**IMD (quintiles)**					
Q1 (least deprived)	84,291 (26.3%)	1087 (20.2%)	239 (6.8%)	658 (23.2%)	193 (18.3%)
Q2	76,334 (23.8%)	1118 (20.8%)	371 (10.5%)	550 (19.4%)	196 (18.6%)
Q3	67,834 (21.2%)	1173 (21.8%)	600 (17.0%)	554 (19.5%)	218 (20.7%)
Q4	52,990 (16.5%)	1051 (19.5%)	1043 (29.5%)	574 (20.2%)	238 (22.6%)
Q5 (most deprived)	38,964 (12.2%)	950 (17.7%)	1286 (36.3%)	504 (17.7%)	210 (19.9%)
**Smoking status**					
Non-smoker	162,934 (50.9%)	4140 (77.0%)	2443 (69.0%)	1701 (59.9%)	616 (58.4%)
Smoker	48,797 (15.2%)	552 (10.3%)	475 (13.4%)	492 (17.3%)	200 (19.0%)
Former smoker	108,682 (33.9%)	687 (12.8%)	621 (17.5%)	647 (22.8%)	239 (22.7%)
**Death**					
Age at death (mean, SD)	80.7 (±11.6)	72.1 (±13.6)	71.8 (±14.8)	74.2 (±15.2)	70.8 (±15.5)
Premature death (Death before age 75)	24,883 (7.8%)	491 (9.1%)	352 (9.9%)	233 (8.2%)	87 (8.2%)
**Cancer site**					
Breast	52,133 (16.3%)	1354 (25.2%)	738 (20.9%)	625 (22.0%)	242 (22.9%)
Prostate	28,157 (8.8%)	480 (8.9%)	812 (22.9%)	185 (6.5%)	109 (10.3%)
Colorectal	23,049 (7.2%)	430 (8.0%)	250 (7.1%)	206 (7.3%)	73 (6.9%)
Bladder	14,439 (4.5%)	251 (4.7%)	93 (2.6%)	121 (4.3%)	39 (3.7%)
Cervical	13,916 (4.3%)	204 (3.8%)	219 (6.2%)	190 (6.7%)	105 (10.0%)
Lung	6938 (2.2%)	168 (3.1%)	74 (2.1%)	67 (2.4%)	24 (2.3%)
All other cancers	181,781 (56.7%)	2922 (54.3%)	1353 (38.2%)	1445 (50.9%)	463 (43.9%)
**Number of comorbidities (n, %)**					
0–1	9841 (3.1%)	230 (4.3%)	160 (4.5%)	194 (6.8%)	61 (5.8%)
2	24,034 (7.5%)	558 (10.4%)	375 (10.6%)	384 (13.5%)	136 (12.9%)
3	32,535 (10.2%)	651 (12.1%)	451 (12.7%)	415 (14.6%)	170 (16.1%)
4	36,156 (11.3%)	637 (11.8%)	461 (13.0%)	366 (12.9%)	136 (12.9%)
5 or more	36,040 (11.2%)	609 (11.3%)	425 (12.0%)	344 (12.1%)	125 (11.8%)

## Data Availability

Restrictions apply to the availability of these data. Data were obtained from CPRD (study protocol 21_000345) and are available at https://www.cprd.com/data-access with the permission of CPRD’s Independent Scientific Advisory Committee for the Medicines and Healthcare Products Regulatory Agency.

## Appendix A

**Table A1:** The 28 cancers included in the “all-cancers combined” analysis. List of cancer sites that were investigated.

Cancer Sites
Hodgkin lymphoma
2. Leukaemia
3. Multiple myeloma and malignant plasma cell neoplasms
4. Non-Hodgkin lymphoma
5. Primary malignancy: biliary tract
6. Primary malignancy: bladder
7. Primary malignancy: bone and articular cartilage
8. Primary malignancy: brain, other CNS, and intracranial
9. Primary malignancy: breast
10. Primary malignancy: cervical
11. Primary malignancy: colorectal and anus
12. Primary malignancy: kidney and ureter
13. Primary malignancy: liver
14. Primary malignancy: lung and trachea
15. Primary malignancy: malignant melanoma
16. Primary malignancy: mesothelioma
17. Primary malignancy: oesophageal
18. Primary malignancy: ovarian
19. Primary malignancy: pancreatic
20. Primary malignancy prostate
21. Primary malignancy: stomach
22. Primary malignancy: testicular
23. Primary malignancy: thyroid
24. Primary malignancy: uterine
25. Primary malignancy: oro-pharyngeal
26. Primary malignancy: other
27. Primary malignancy: other skin and subcutaneous tissue
28. Secondary malignancy and metastasis

**Table A2:** Odds of comorbidity by ethnicity adjusted for age, sex, BMI, IMD, and smoking status.

	White	Asian	Black	Other
Comorbidity		Adjusted Odds Ratio (95% CI)	Adjusted Odds Ratio (95% CI)	Adjusted Odds Ratio (95% CI)
Anaemia	Ref	2.33 (2.19–2.48)	1.97 (1.83–2.13)	1.12 (1.02–1.24)
Anxiety and phobia	Ref	0.53 (0.49–0.57)	0.45 (0.41–0.49)	0.66 (0.60–0.72)
Asthma	Ref	1.19 (1.11–1.27)	0.83 (0.76–0.91)	0.79 (0.71–0.87)
Atrial fibrillation	Ref	0.68 (0.62–0.75)	0.53 (0.47–0.61)	0.81 (0.71–0.92)
Chronic kidney disease	Ref	1.47 (1.36–1.59)	1.55 (1.42–1.70)	0.85 (0.76–0.96)
Constipation	Ref	1.5 (1.41–1.59)	1.33 (1.24–1.44)	1.02 (0.94–1.11)
Coronary artery disease	Ref	1.7 (1.59–1.83)	0.77 (0.69–0.85)	0.94 (0.84–1.05)
Depression	Ref	0.62 (0.58–0.66)	0.54 (0.50–0.59)	0.71 (0.65–0.77)
Dermatitis	Ref	1.31 (1.24–1.39)	0.78 (0.72–0.84)	0.88 (0.81–0.96)
Diabetes	Ref	4.41 (4.13–4.70)	2.13 (1.96–2.32)	1.75 (1.58–1.94)
Hearing loss	Ref	0.84 (0.78–0.91)	0.54 (0.48–0.60)	0.77 (0.70–0.86)
Hypertension	Ref	1.76 (1.55–2.00)	2.32 (1.95–2.76)	1.12 (0.93–1.36)
Obesity	Ref	1.29 (1.19–1.40)	0.97 (0.88–1.07)	0.94 (0.83–1.06)
Osteoarthritis	Ref	1.04 (0.97–1.11)	0.83 (0.77–0.90)	0.71 (0.64–0.78)
Painful condition	Ref	1.64 (1.46–1.85)	1.08 (0.92–1.28)	0.80 (0.67–0.97)

**Table A3:** Odds of comorbidity by IMD adjusted for age, sex, BMI, ethnicity, and smoking status.

Comorbidity	IMD 1	IMD 2	IMD 3	IMD 4	IMD 5 (Least Affluent)
		Adjusted Odds Ratio (95% CI)	Adjusted Odds Ratio (95% CI)	Adjusted Odds Ratio (95% CI)	Adjusted Odds Ratio (% CI)
Anaemia	Ref	1.03 (1.00–1.06)	1.06 (1.03–1.09)	1.12 (1.09–1.15)	1.28 (1.24–1.32)
Anxiety and phobia	Ref	1.07 (1.02–1.13)	1.10 (1.04–1.16)	1.25 (1.18–1.33)	1.32 (1.23–1.41)
Asthma	Ref	1.06 (1.04–1.08)	1.12 (1.09–1.14)	1.25 (1.22–1.28)	1.55 (1.51–1.59)
Atrial fibrillation	Ref	0.98 (0.96–1.00)	1.00 (0.97–1.02)	1.01 (0.98–1.03)	1.07 (1.04–1.10)
Chronic kidney disease	Ref	1.08 (1.05–1.10)	1.15 (1.13–1.18)	1.28 (1.24–1.31)	1.57 (1.52–1.62)
Constipation	Ref	1.01 (0.99–1.04)	1.03 (1.00–1.06)	1.06 (1.03–1.09)	1.16 (1.12–1.19)
Coronary artery disease	Ref	1.06 (1.04–1.09)	1.13 (1.10–1.16)	1.25 (1.22–1.29)	1.45 (1.41–1.50)
Depression	Ref	1.14 (1.08–1.20)	1.26 (1.20–1.33)	1.53 (1.45–1.62)	1.97 (1.85–2.10)
Dermatitis	Ref	1.03 (1.00–1.05)	1.09 (1.06–1.11)	1.17 (1.14–1.20)	1.35 (1.31–1.39)
Diabetes	Ref	1.07 (1.03–1.10)	1.12 (1.09–1.16)	1.27 (1.23–1.31)	1.40 (1.36–1.45)
Hearing loss	Ref	1.06 (1.03–1.08)	1.17 (1.15–1.20)	1.29 (1.26–1.32)	1.52 (1.48–1.56)
Hypertension	Ref	1.06 (1.03–1.10)	1.16 (1.13–1.20)	1.22 (1.18–1.26)	1.21 (1.17–1.25)
Obesity	Ref	1.08 (1.05–1.10)	1.16 (1.13–1.19)	1.28 (1.25–1.32)	1.52 (1.48–1.57)
Osteoarthritis	Ref	1.04 (1.01–1.06)	1.07 (1.05–1.10)	1.14 (1.11–1.16)	1.24 (1.21–1.27)
Painful condition	Ref	0.91 (0.90–0.93)	0.91 (0.89–0.93)	0.87 (0.85–0.89)	0.91 (0.88–0.93)

**Table A4:** Adjusted hazard ratio (HR) of mortality by broad ethnic groups—adjusted for age, sex, BMI, smoking status, and IMD (significant HR are in bold).

	White	Asian HR (95% CI)	Black HR (95% CI)	Other HR (95% CI)
All cancers combined	Ref	**1.31 (1.23–1.39)**	**1.48 (1.38–1.59)**	**1.10 (1.00–1.19)**
Breast	Ref	1.12 (0.97–1.29)	**1.78 (1.52–2.10)**	1.07 (0.88–1.31)
Prostate	Ref	1.11 (0.91–1.34)	**1.23 (1.06–1.42)**	1.07 (0.79–1.44)
Colorectal	Ref	1.11 (0.92–1.35)	**1.55 (1.23–1.95)**	1.01 (0.78–1.32)
Bladder	Ref	1.12 (0.85–1.47)	1.18 (0.75–1.85)	0.89 (0.59–1.34)
Cervical	Ref	**1.73 (1.02–2.95)**	1.59 (0.95–2.66)	**1.90 (1.19–3.03)**
Lung	Ref	**1.81 (1.44–2.28)**	1.38 (0.95–2.00)	0.92 (0.58–1.46)

**Table A5:** Adjusted hazard ratio of mortality by socioeconomic status—adjusted for age, sex, BMI, smoking status, and ethnicity (significant HR are in bold).

	IMD 1 (Most Affluent)	IMD 2 HR (95% CI)	IMD 3 HR (95% CI)	IMD 4 HR (95% CI)	IMD 5 HR (95% CI)
All cancers combined	Ref	**1.09 (1.07–1.11)**	**1.10 (1.08–1.12)**	**1.14 (1.12–1.1)**	**1.28 (1.25–1.30)**
Breast	Ref	**1.08 (1.03–1.13)**	**1.14 (1.08–1.20)**	**1.13 (1.07–1.19)**	**1.28 (1.21–1.36)**
Prostate	Ref	1.05 (0.99–1.11)	**1.11 (1.05–1.18)**	**1.15 (1.08–1.22)**	**1.18 (1.10–1.27)**
Colorectal	Ref	**1.09 (1.03–1.16)**	**1.08 (1.01–1.14)**	**1.07 (1.00–1.14)**	**1.22 (1.13–1.30)**
Bladder	Ref	**1.15 (1.06–1.25)**	**1.17 (1.08–1.27)**	**1.18 (1.08–1.28)**	**1.25 (1.14–1.37)**
Cervical	Ref	1.11 (0.91–1.35)	1.08 (0.89–1.31)	1.09 (0.90–1.32)	**1.31 (1.09–1.59)**
Lung	Ref	0.97 (0.87–1.07)	0.90 (0.81–1.00)	1.00 (0.91–1.11)	0.96 (0.87–1.07)
